# Direct Lithium Extraction from a Complex Acidic Brine Using Aluminum Hydroxide

**DOI:** 10.1021/acsomega.5c12622

**Published:** 2026-05-08

**Authors:** Alan Piao, Jianjun Chen, Chengi Hung, Yuqing Fu, Nastaran Shojarazavi, Bryan M. Wong, Juchen Guo

**Affiliations:** † Oak Park High School, 899 Kanan Rd, Oak Park, California 91377, United States; ‡ Department of Chemical and Environmental Engineering, 248718 University of California, Riverside, California 92521, United States; § Department of Chemistry, University of California, Riverside, California 92521, United States

## Abstract

Aluminum hydroxide (Al­(OH)_3_) is evaluated as a sorbent to directly extract lithium (Li) from a complex, acidic brine containing low-concentration Li^+^ and multiple metal cations with high concentration, including sodium (Na^+^), potassium (K^+^), calcium (Ca^2+^), manganese (Mn^2+^), and iron (Fe^2+^). Extraction experiments indicate that Al­(OH)_3_ can extract all cations except Ca^2+^ in the brine neutralized by excess Al­(OH)_3_. Higher reaction temperatures enhance the extraction efficiency but reduce the cation selectivity. When the pH of brine is adjusted to 11, all Mn^2+^ and Fe^2+^ cations precipitate out of the brine, and the alkaline condition may facilitate the formation of Ca–Al layered double hydroxide (LDH) structure in competition with the formation of Li–Al LDH. To recover the extracted Li, the extraction product is heat-treated to convert LDH to Boehmite (γ-AlO­(OH)) that has a weaker affinity with Li^+^. Simple room-temperature water rinsing applied to the Boehmite demonstrates higher recovery of Li and separation of Li from other undesirable cations.

## Graphical abstract

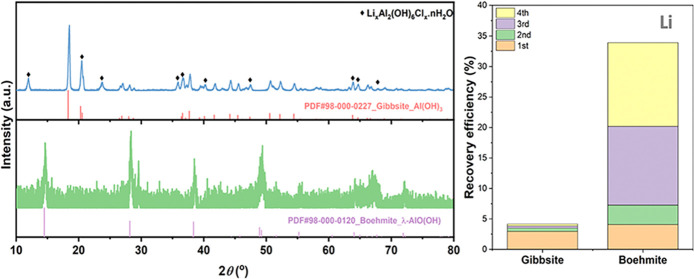

## Introduction

Lithium (Li)’s importance as a natural resource is rapidly growing, primarily driven by increasing demand for Li-ion batteries (LIBs). LIBs are critical enablers for electric vehicles, personal electronics, grid energy storage, and more.^1^ In 2021, global consumption of Li was 93,000 MT, of which 74% was contributed by the LIB industry.^2^ It is projected that, in 2030, global Li demand will be more than 2 million MT, of which the LIB industry will account for 95%. Currently, Li is primarily sourced from two methods: mining solid Li ores and extraction from Li-rich brines. Terrestrial mining, targeting minerals such as spodumene (LiAl­(SiO_3_)_2_) and petalite (LiAlSi_4_O_10_), presents a consistently productive and relatively straightforward solution. However, rapid depletion of ores, high energy demand for extraction and processing, and inherent ecological concerns make this method of extraction undesirable in the long term.^2^ Extraction from Li-rich brine, estimated to contain 66% of the world’s total Li reserves, is a more cost-effective and energy-efficient method.^3^ Currently, conventional solar evaporation, where brine is first pumped into evaporation ponds to concentrate before Li is separated through selective precipitation of undesired ions with various chemical reagents, is the most common industrial technique, sporting low operational costs and energy investment, due to its reliance on solar energy. This method, however, still has several notable flaws. Hazardous waste, possible environmental damage, and its time-consuming nature are all concerning realities for the practice. Additionally, the arid climate necessary to support such methods precludes many possibilities for development.^2,3^ The focal method of this paper, direct lithium extraction (DLE) by adsorption/absorption with aluminum hydroxide (Al­(OH)_3_), presents a promising alternative to these existing processes, especially with respect to geothermal brine resources.^4^

DLE consists of selectively extracting Li^+^ cations from brine. Several technologies, including ion exchange, solvent extraction, membranes, and adsorption, have been proposed, each with its own benefits and challenges. Ion exchange utilizes manganese- and titanium-based adsorbents to achieve high lithium extraction performance with a relatively low rate of contaminants. However, this process falls short due to high waste formation, environmental concerns, high operating expenses, and the use of large quantities of acid, among other concerns.^3^ Solvent extraction proposes an efficient and continuous process at the critical disadvantages of environmental concerns, high waste production, and uncompetitive capital expenditures.^2,3^ Extraction using membranes involves selective transport through nanopores, a continuous process that produces fewer impurities. However, the technology currently suffers from major cost concerns and critical issues with membrane fouling as well as the additional downside of requiring significant pretreatment.^3^ As of present, adsorption through Al-based sorbents, specifically Al­(OH)_3_, shows significant promise for industrially applicable DLE.^5,6^

Adsorption-type extraction of Li using Al­(OH)_3_ has been demonstrated to display high extraction efficiency and high selectivity for Li^+^ ions over competing ions, making it an especially viable choice for industrial DLE.^7–9^ Extraction using this method also does not require acid, uses an inexpensive reagent (Al­(OH)_3_), and has low environmental impact.^5^ Al­(OH)_3_ forms Li_*x*_Al_2_(OH)_6_Cl_*x*_·*n*H_2_O with a layered double hydroxide (LDH) structure (Li–Al LDH) when in aqueous lithium chloride (LiCl) solution. The LiCl intercalates into the plate-like two-dimensional aluminum hydroxide layers, with Li^+^ integrated into the unoccupied octahedral sites.^10^ Al­(OH)_3_ demonstrates high selectivity for Li^+^ due to the small ion size of Li^+^ compared with competing monovalent cations in solution, such as sodium (Na) and potassium (K) cations, causing the separation of Li^+^ and forming the central mechanism of this method. Al-dhawi et al. demonstrated a maximum recovery rate of 94% using a simple brine of 10 wt % LiCl and 90 wt % distilled water.^5^ Jayanthi et al. demonstrated 82% Li^+^ adsorption by stoichiometric amorphous Al­(OH)_3_ with high selectivity against competing Na^+^ and K^+^ ions.^6^ Majority of the previous studies on the feasibility of Al­(OH)_3_ for adsorption-type DLE have used simple brines except a few studies on simulated geothermal brines.^11–13^ Critically, DLE via adsorption using Al­(OH)_3_ has been demonstrated to be highly dependent on initial Li concentration, pH, type of anions, and adsorbent dosage.^2,6,14^ Therefore, investigations must be conducted with more representative feed stocks. The focus of this study is to examine the mechanism and potential complexity of DLE using Al­(OH)_3_ from a complex acidic simulant representative of geothermal brine. This study demonstrates the effect of pH on the solubility of transition-metal ions (Fe^2+^ and Mn^2+^) and the selectivity of Ca^2+^. Additionally, thermal conversion of the extraction product, i.e., Li–Al LDH, to Boehmite is demonstrated as an effective method to recover Li.

## Experimental Section

The complex acidic brine simulating a geothermal Li resource is composed of LiCl, sodium chloride (NaCl), potassium chloride (KCl), calcium chloride (CaCl_2_), iron­(II) chloride (FeCl_2_), and manganese­(II) chloride (MnCl_2_) and acidified by boric acid (H_3_BO_3_). The specific contents are listed in Table 1. The brine is prepared in degassed deionized water to avoid Fe^2+^ oxidation. A fresh brine sample was prepared for each experiment.

**Table 1:** Composition of the Synthetic Brine

composition	mass (g)	conc. of cation (mg mL^–1^)
NaCl	70.76	67.58
KCl	15.99	20.37
LiCl	0.722	0.29
CaCl_2_·2H_2_O	61.21	40.52
FeCl_2_·4H_2_O	3.05	2.08
MnCl_2_·4H_2_O	3.86	2.60
H_3_BO_3_	1.26	
H_2_O	343.15	

### Li Extraction from Pristine Brine

Anhydrous Gibbsite Al­(OH)_3_ (Sigma-Aldrich) was mixed in 15 mL of pristine brine with 4 different Li^+^/Al­(OH)_3_ mass ratios at 1:5, 1:10, 1:20, and 1:40, and the slurry was continuously stirred at 3 different temperatures of 55 °C, 75 °C, and 95 °C for 48 h. Each experiment was triplicated. The solid in the slurry was separated via centrifugation and subsequently rinsed with 45 mL of deionized water through agitation by stirring. The solid that separated after rinsing was defined as the extraction product. The elemental content in the combined supernatants from the two steps above was analyzed through inductively coupled optical emission spectroscopy (ICP-OES) to determine the extraction efficiency of different cations.

### Li Extraction from pH-Adjusted Brine

The pH of the pristine brine is 3.6, from which brine samples with pH values at 4.0, 4.9, 5.9, 6.5, 7.2, 7.5, 8.5, 9, 10.5, 11.1, and 12 were obtained by adding different amounts of 5 M potassium hydroxide (KOH) solution in deionized water. The elemental compositions of all pH-adjusted brines (including the pristine brine) were analyzed with ICP-OES to determine the relationship between the pH value and the ion concentration. The iron­(II) hydroxide (Fe­(OH)_2_) and manganese­(II) hydroxide (Mn­(OH)_2_) precipitates were removed through centrifugation from the pH-adjusted brine at pH = 11.1. This brine was divided into two equal portions, and the pH value of one of them was adjusted to pH 4.5 using diluted hydrochloric acid (HCl). Li extraction was performed using both brine samples with a 1:10 Li^+^/Al­(OH)_3_ mass ratio at 75 °C. The reaction and the extraction efficiency measurement were the same as the pristine brine. Each experiment was triplicated.

### Li Recovery from the Extraction Products

The extraction product from the brine at pH 11.1 was used for Li recovery. The formation of Li–Al LDH was confirmed by X-ray diffraction (XRD). Two methods were used to recover Li: (1) The extraction product was directly rinsed with deionized water for 4 cycles with 3 mL per cycle at room temperature. The ion content in each rinsing solution was analyzed with ICP-OES to obtain the recovery efficiency. (2) The extraction product was heat-treated at 300 °C for 20 min in air to convert Li–Al LDH to a Boehmite structure that was confirmed with XRD. The obtained Boehmite was rinsed with deionized water for 4 cycles with 3 mL per cycle at room temperature. The ion content in each rinsing solution was analyzed with ICP-OES to obtain the recovery efficiency.

### Computational Study of Selectivity

All density functional theory (DFT) calculations were carried out using the Vienna Ab initio Simulation Package (VASP)^15^ with planewave basis sets and a kinetic energy cutoff of 650 eV. Projector augmented wave (PAW) potentials were used to describe the electron–ion interactions.^16,17^ The PBE functional was used along with DFT-D3 dispersion correction to account for van der Waals interactions.^18,19^ Force tolerance for all calculations was set to 0.00001 eV/Å. Crystal structures for LiAl_2_(OH)_6_Cl were taken from the literature^20^;^20^ the corresponding Na/K analogs were constructed by direct cation substitution. During optimization, both the atomic coordinate and the cell are allowed to relax. Distorted Al­(OH)_3_ structures used to evaluate distortion and intercalation energies were generated from optimized MAl_2_(OH)_6_Cl structures by removing the M and Cl atoms. Subsequent geometry optimizations were performed with fixed lattice parameters. The distortion and intercalation energies were calculated using the following reactions

1 $$2\mathrm{Al}{\left(\mathrm{OH}\right)}_{3}\to 2\mathrm{Al}{\left(\mathrm{OH}\right)}_{3}\;\left(\mathrm{distorted}\right)$$

2 $$2\mathrm{Al}{\left(\mathrm{OH}\right)}_{3}\;\left(\mathrm{distorted}\right)+\mathrm{MCl}\to M{\mathrm{Al}}_{2}{\left(\mathrm{OH}\right)}_{6}\mathrm{Cl}$$

The total energy *E*_total_ = *E*_distortion_ + *E*_intercalation._

## Results and Discussion

The key features of the brine in this study include the two magnitude lower concentration of Li^+^ than those of Na^+^, K^+^, and Ca^2+^, the content of multiple divalent metal cations, and the acidity. The low LiCl concentration relative to other cations and the acidity of the brine are not in favor of Li–Al LDH formation.^21,22^ Meanwhile, all divalent cations in this brine, Ca^2+^, Fe^2+^, and Mn^2+^, are capable to form LDH with Al­(OH)_3_ with a general formula of [M­(II)_1‑*x*_Al_*x*_(OH)_2_]­Cl_*x*_·*n*H_2_O (0 < *x* < 1).^23^ Previous studies indicate that the formation of these LDH compounds generally prefers alkaline conditions with different optimal pH values.^20–24^ The formation of Ca–Al LDH prefers a strong alkaline solution with pH at ∼11 ^24,25^ ;^24,25^ the formation of Mn–Al LDH prefers a pH between 9 and 10.5 ^26,27^ ;^26,27^ and the formation of Fe–Al LDH prefers a lower pH between 8.8 and 9.5.^27,28^

To shed light on the effect of these complications on Li extraction, the efficiency to extract Li directly from this pristine brine was tested. The Li_*x*_Al_2_(OH)_6_Cl_*x*_·*n*H_2_O LDH has a theoretical maximum Li^+^ uptake with the formula of LiAl_2_(OH)_6_Cl·*n*H_2_O, in which the Li^+^/Al­(OH)_3_ mass ratio is 1:11. Therefore, 4 different Li^+^/Al­(OH)_3_ mass ratios at 1:5, 1:10, 1:20, and 1:40 were tested. Heating the brine (typically between 40 and 95 °C) has been proven to be necessary to effectively extract Li using Al­(OH)_3_.^2^ Therefore, to understand the effect of temperature on the extraction, the reactions were performed at three different temperatures, including 55 °C, 75 °C, and 95 °C. It is worth noting that the pH of the brine after adding Al­(OH)_3_, in any Li^+^/Al­(OH)_3_ ratio, was increased to a pH value around 5.5. Therefore, the extraction reactions actually took place under slightly acidic conditions. The extraction efficiency (defined as the ratio of the ion mass in the extraction product to the ion mass in the pristine brine) is displayed in Figure 1a. A distinct observation from the results is that the Ca^2+^ ion was not extracted by Al­(OH)_3_ in any condition, which is consistent with the prior knowledge that Ca–Al LDH formation requires a strong alkaline condition. The extraction of K^+^ is minimal: K^+^ only consistently appears in the extraction product from the reactions at 95 °C. However, a significant percentage of Na^+^ cations were picked up by the Al­(OH)_3_ sorbent under all conditions. The total energy required to form Li–Al, Na–Al, and K–Al LDH compounds was calculated with the DFT method and listed in Table 2. The result indicated that the selectivity follows the trend of Li^+^ > Na^+^ ≫ K^+^, which is consistent with the previous report.^29^

**Figure 1:**
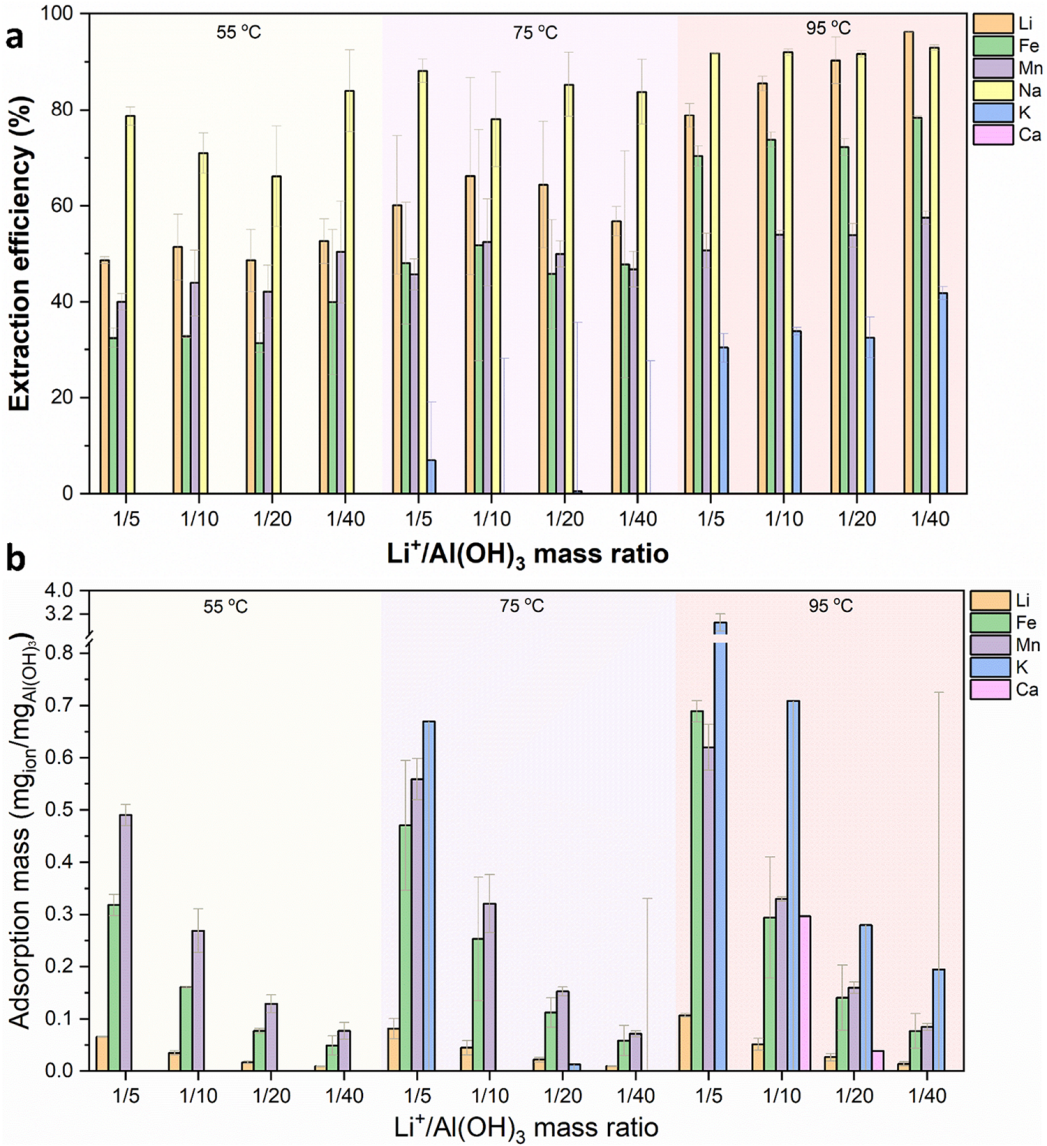
(a) Extraction efficiency of all metal ions and (b) ion mass uptake per unit mass of Al­(OH)_3_ (without Na^+^) at different Li^+^/Al­(OH)_3_ mass ratios and temperatures.

**Table 2:** Calculated Energy Required to Form LDH between Al­(OH)_3_ and LiCl, NaCl, and KCl

cation	*E*_distortion_ (eV)	*E*_intercalation_ (eV)	*E*_total_ (eV)
Li^+^	1.03	–1.51	–0.48
Na^+^	0.94	–0.63	0.32
K^+^	1.21	0.42	1.63

There is a clear general trend that the extraction efficiencies of almost all ions increase with increasing temperature. The extraction results can also be expressed as extraction mass (defined as extracted ion mass per gram of Al­(OH)_3_), displayed in Figure 1b. The extraction mass of Na^+^ is overwhelming, higher than 2 mg/mg_Al(OH)3_ under any extraction condition; therefore, it is removed from Figure 1b for clarity. In general, the adsorbed metal ions per unit mass of Al­(OH)_3_ decrease as the Li^+^/Al­(OH)_3_ ratio decreases. Considering the Li extraction efficiency, extraction purity, energy consumption, and the usage of Al­(OH)_3_, it was decided that the further extraction tests would be performed with the 1:10 Li^+^/Al­(OH)_3_ mass ratio at 75 °C.

Although Ca^2+^ cations were not adsorbed by Al­(OH)_3_, it is interesting that both Fe^2+^ and Mn^2+^ cations were extracted by Al­(OH)_3_. As aforementioned, the formation of divalent cation Al LDH is not through intercalation as Li^+^ does; Instead, they form via the dissolution-and-reprecipitation route; thus, their formation requires an alkaline solution of aluminate species. The mixture of brine and Al­(OH)_3_ is slightly acidic so that Fe–Al LDH and Mn–Al LDH are unlikely to form. On the other hand, previous studies indicated transition-metal ions could be adsorbed on the surface of LDH compounds due to surface charge or surface-induced Fe­(II) oxidation.^30–32^ The Fe^2+^ and Mn^2+^ ions in the brine can be removed to increase the pH of the brine to avoid the undesirable uptake. The ion concentrations as a function of pH of the brine are measured as displayed in Figure 2. The top panel shows the concentrations of high-concentration ions, including Na^+^, K^+^, and Ca^2+^, have almost no changes except a slight increase of K^+^, since the pH was increased through adding KOH solution. The concentration of the low-concentration ions as a function of pH, shown in the lower panel in Figure 2, indicates that all Fe^2+^ ions precipitate out of the brine at a pH of 8.5, and all Mn^2+^ ions precipitate out at a pH of 10.5, while the concentration of Li^+^ seems unchanged.

**Figure 2:**
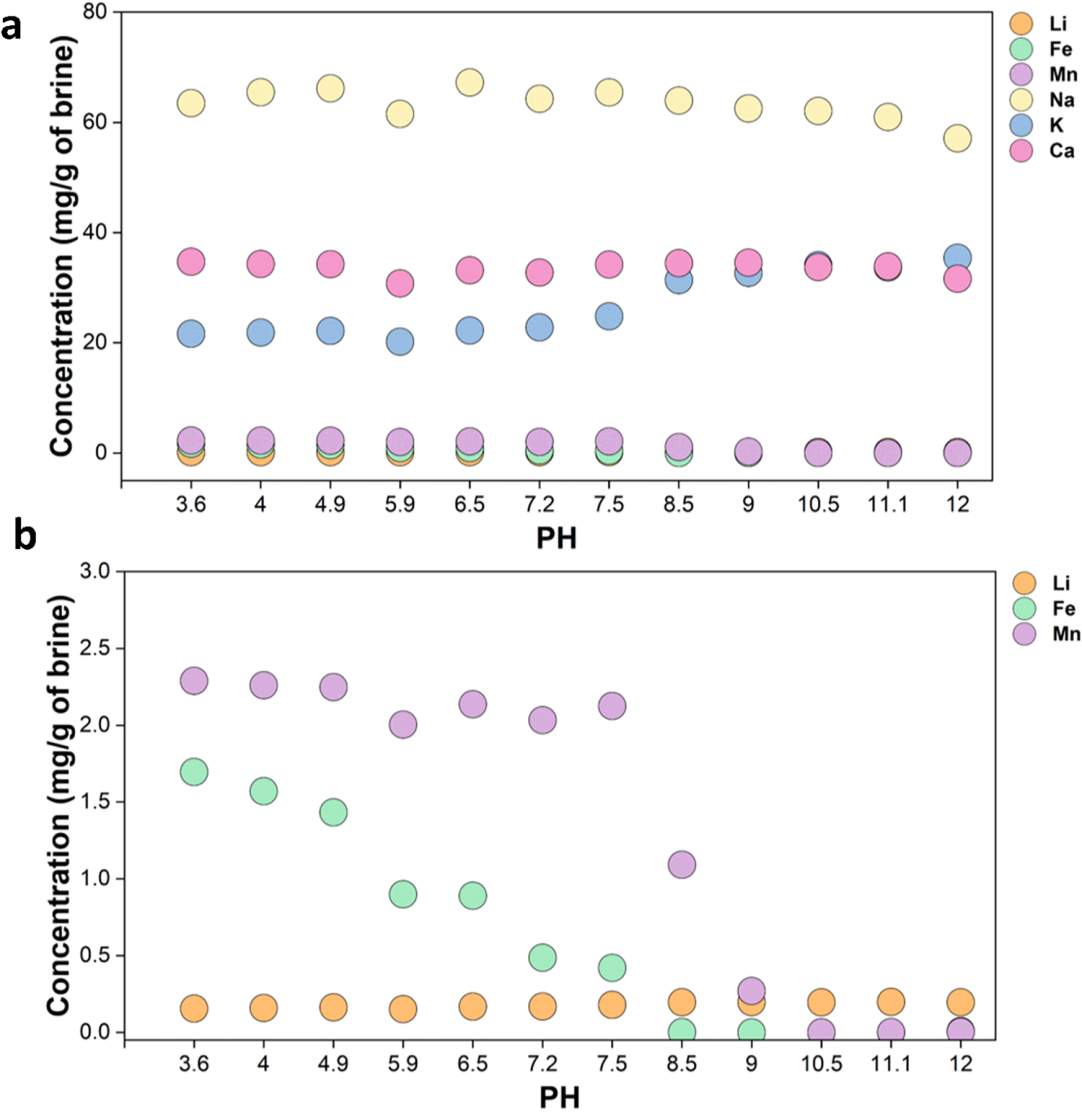
(a) Concentration of all ions in the brine as a function of pH value. (b) The concentration of Li^+^, Fe^2+^, and Mn^2+^ as a function of pH value.

The extraction experiments were subsequently performed using the brine with the pH value adjusted to 11.1. To understand the effect of pH on extraction efficiency, the same experiment was performed with the brine where the pH was adjusted back to an acidic condition at pH = 4.5.

The results in Figure 3 indicate that Li^+^ and Na^+^ cations were picked up by Al­(OH)_3_ from both brines as well as K^+^ and Ca^2+^ cations. The Li^+^ extraction efficiency from the brine at pH 4.5 is 66% and that from the brine at pH 11 is higher at 75%. When the extraction efficiency results are converted to extracted mass per unit mass of Al­(OH)_3_, as shown in Figure 3b, it shows that 0.05 mg of Li^+^ was extracted per mg of Al­(OH)_3_, which is approximately half of the theoretical extraction, from the brine with pH = 11. The extraction of Na^+^ per mg of Al­(OH)_3_ is significant, and either the Na^+^ extraction efficiency or the amount of Na^+^ per unit mass of Al­(OH)_3_ is almost identical to those from the pristine brine. This result indicates that Na^+^ uptake is likely via true surface physical adsorption, and its significant uptake is unavoidable if the concentration is high. Both K^+^ and Ca^2+^ were also extracted, but with much lower efficiency than those of Li^+^ and Na^+^. However, the mass of K^+^ and Ca^2+^ per mg of Al­(OH)_3_ is substantial. It is expected that Ca–Al LDH could form in the pH 11 brine due to its alkaline condition; but Ca^2+^ ions were also adsorbed by Al­(OH)_3_ in the brine of pH 4.5, in which Ca–Al LDH compound cannot be formed. Therefore, the majority of Ca^2+^ in Al­(OH)_3_ can be through physical adsorption.

**Figure 3:**
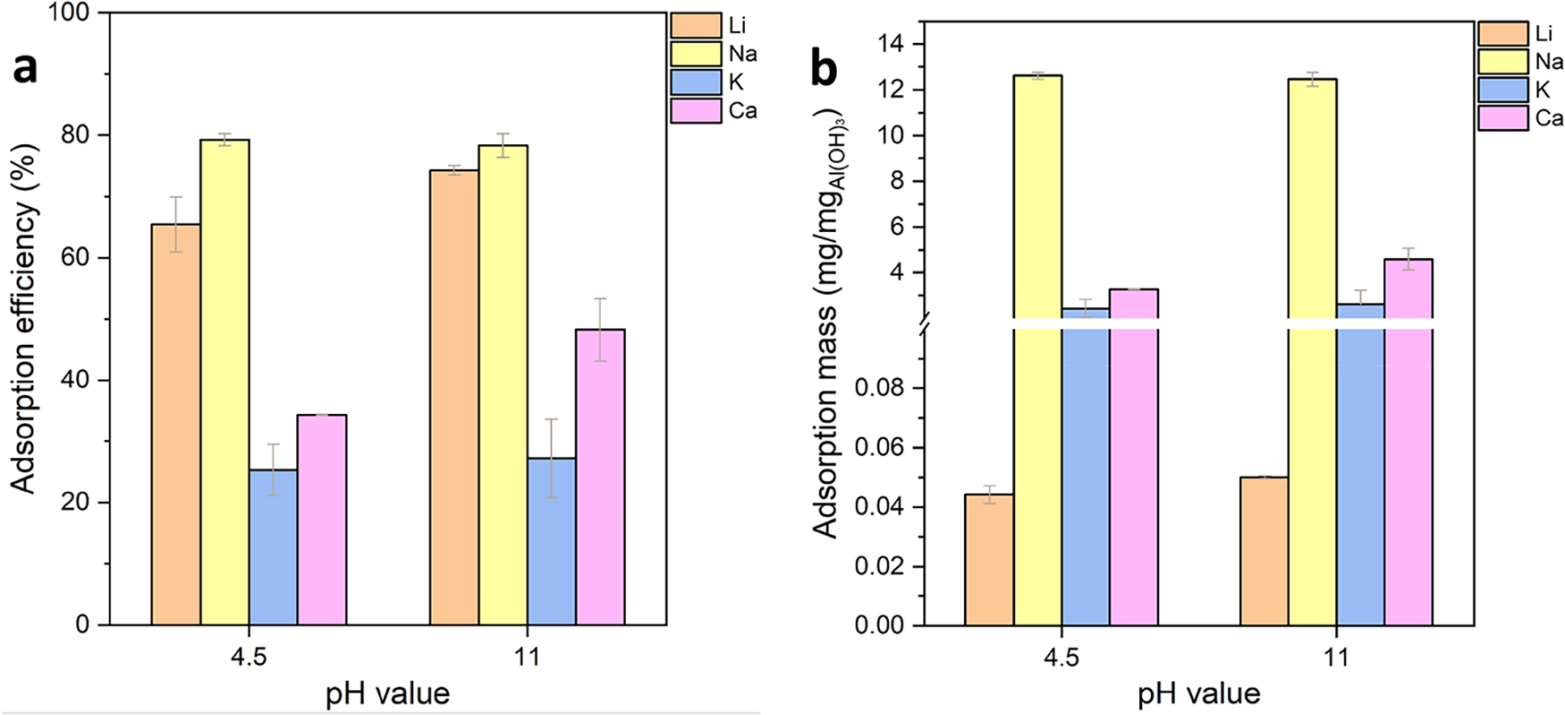
(a) Ion extraction efficiency and (b) mass uptake from brine samples after Fe and Mn removal at two different pH levels with a 1:10 Li^+^/Al­(OH)_3_ mass ratio at 75 °C.

Recovery of Li from the extraction product obtained from the brine at pH 11 by rinsing with room-temperature deionized water was investigated. The XRD pattern of the extraction product (Figure 4a) confirms the formation of crystalline Li–Al LDH in the extraction product,^33^ alongside the unreacted Gibbsite Al­(OH)_3_. We hypothesize that rinsing Li^+^ directly from Li–Al LDH can be ineffective due to the strong affinity between Li^+^ and the Al_2_(OH)_6_ framework. Therefore, we heat-treated the extraction product at 300 °C for 20 min to convert the LDH structure to Boehmite (γ-AlO­(OH)) structure, which is confirmed by the XRD pattern of the heat-treated product in Figure 4b. The XRD pattern also indicates the obtained Boehmite is poorly crystalline, which is beneficial to Li recovery. The hypothesis is that Li^+^ incorporation in defect-rich Boehmite is mostly through superficial intercalation, thus more accessible to removal through rinsing.^34^

**Figure 4:**
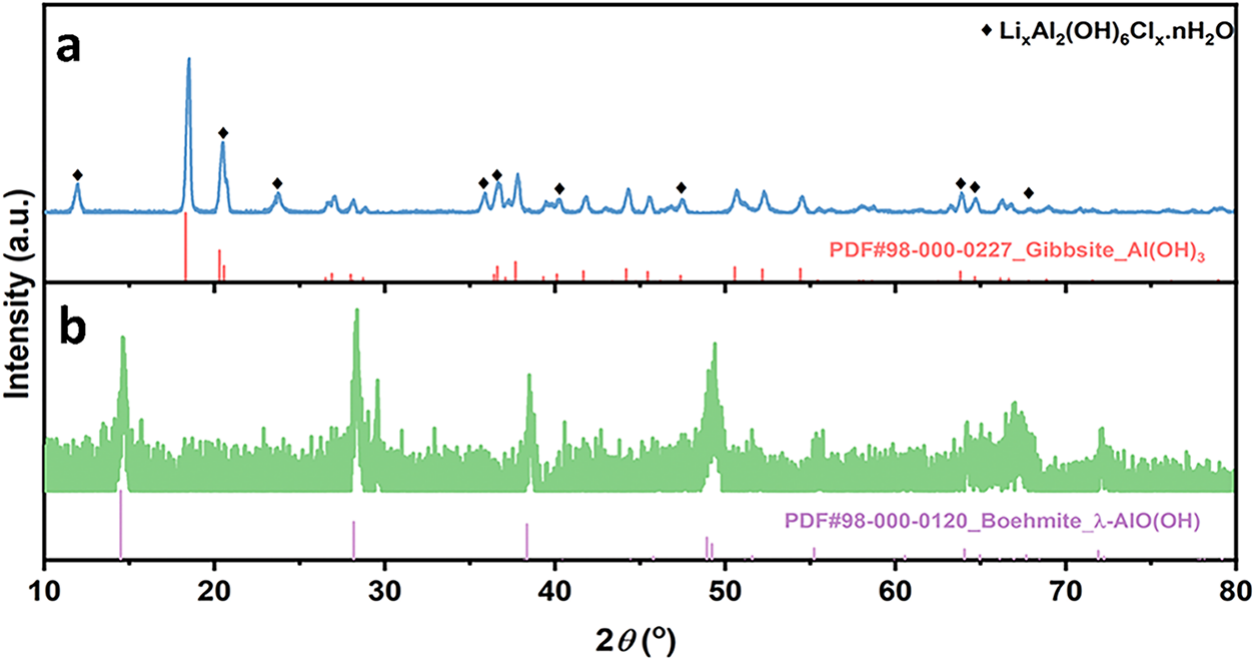
XRD pattern of (a) the pristine extraction product and (b) the heat-treated extraction product.

Li recovery from both the pristine extraction product (denoted as Gibbsite) and the heat-treated extraction product (denoted as Boehmite) was performed through 4 water rinsing cycles using deionized water at room temperature. The recovery efficiency (ratio of cation rinsed out to the amount in the extraction product) is shown in Figure 5. It is clear that more cations can be rinsed out from the Boehmite compound. Particularly, the Li recovery efficiency from the Boehmite was significantly higher than that from the Gibbsite. This result supports the hypothesis that the extraction efficiency is improved with crystal structure conversion. It is significant that the amount of Na^+^, K^+^, and Ca^2+^ that can be rinsed out decreases each cycle, while the amount of Li^+^ rinsed out increases each cycle: 95% of K^+^, 63% of Na^+^, and 20% of Ca^2+^ were rinsed out in the first two cycles, and only 7% of Li^+^ was rinsed out in the first two cycles. On the contrary, only 4% of K^+^, 7% of Na^+^, and 3% of Ca^2+^ were rinsed out in the last two cycles, but 27% of Li was rinsed out in the last two cycles. This trend is clearly in favor of Li recovery. The impurities, including K^+^, Na^+^, and Ca^2+^, can be removed with a controlled water rinsing step with minimal Li loss. Then, Li^+^ can be recovered with extra water rinsing steps with a minimized impurity. This observation also supports that Na^+^ and K^+^ are likely to be adsorbed on the surface of Al­(OH)_3_ (and Boehmite after heat treatment), thus they are faster to be dissolved in water compared with the Li^+^ adsorbed inside LDH (and Boehmite after heat treatment).

**Figure 5:**
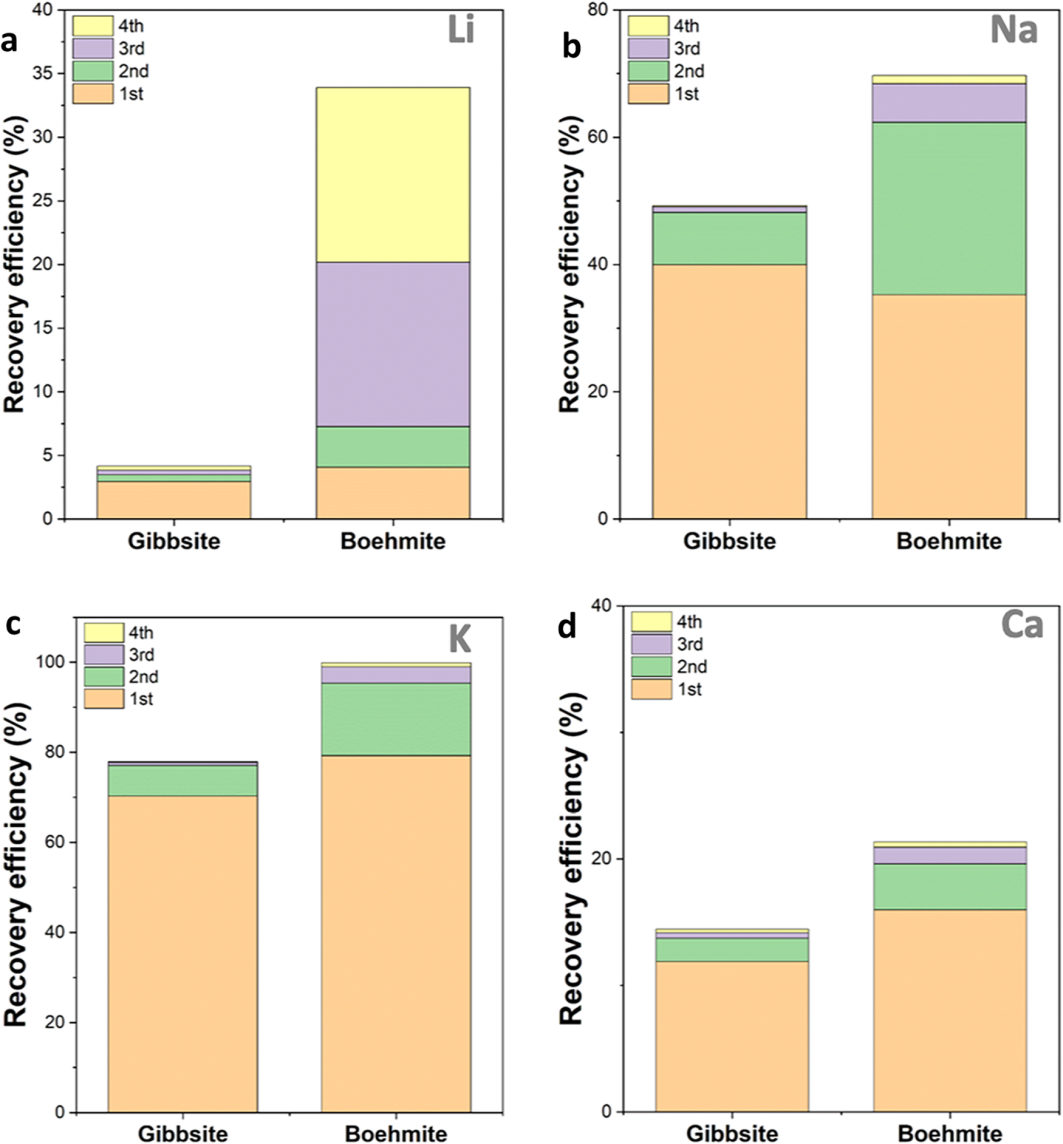
Recovery efficiency of (a) Li, (b) Na, (c) K, and (d) Ca from the pristine (Gibbsite) and heat-treated (Boehmite) extraction product.

## Conclusions

The DLE process in this study is illustrated in Scheme 1. This study demonstrates Al­(OH)_3_ is an effective DLE reagent by forming LDH compound; however, significantly higher concentrations of other cations and acidity of the brine can complexify the DLE process. The concentration of Na^+^ and K^+^ are orders of magnitude higher than that of Li^+^ in the studied brine, thus it is inevitable that considerable amounts of Na^+^ and K^+^ are extracted through physical adsorption. Converting Li–Al LDH to Li-containing γ-AlO­(OH) structure through heat treatment was demonstrated to facilitate Li recovery. Furthermore, the recovery results indicate a natural separation of Li^+^ from the Na^+^ and K^+^ impurity by simple water rinsing. It is still ambiguous if Ca–Al LDH is formed in the pH-adjusted (alkaline) brine and how its formation competes with Li–Al LDH, which are the subjects of future study.

**Scheme 1:**
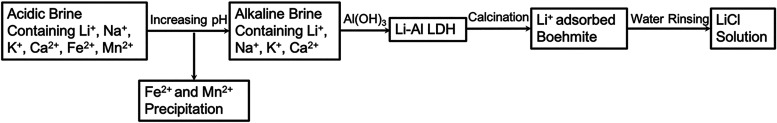
DLE Process from an Acidic Complex Brine in This Study
